# The theta-syllable: a unit of speech information defined by cortical function

**DOI:** 10.3389/fpsyg.2013.00138

**Published:** 2013-03-20

**Authors:** Oded Ghitza

**Affiliations:** Biomedical Engineering, Hearing Research Center, Boston UniversityBoston, MA, USA

**Keywords:** everyday speech, syllabic parsing, cascaded neuronal oscillations, hierarchical window structure, synchronization

## Abstract

A recent commentary (Oscillators and syllables: a cautionary note. Cummins, 2012) questions the validity of a class of speech perception models inspired by the possible role of neuronal oscillations in decoding speech (e.g., Ghitza, 2011; Giraud and Poeppel, 2012). In arguing against the approach, Cummins raises a cautionary flag “from a phonetician's point of view.” Here we respond to his arguments from an auditory processing viewpoint, referring to a phenomenological model of Ghitza (2011) taken as a representative of the criticized approach. We shall conclude by proposing the theta-syllable as an information unit defined by cortical function—an alternative to the conventional, ambiguously defined syllable. In the large context, the resulting discussion debate should be viewed as a subtext of acoustic and auditory phonetics vs. articulatory and motor theories of speech reception.

Anchored at a phonetician viewpoint, a recent commentary (Cummins, 2012) questions the validity of a class of speech perception models inspired by the possible role of neuronal oscillations in decoding speech (e.g., Ghitza, 2011; Giraud and Poeppel, 2012). Cummins' skepticism is in the following three respects: (1) since speech acoustics is all but temporally periodic, speech perception models with oscillations at the core are unfounded, (2) oscillation-based models do not have the structure necessary to decode the rich spectro-temporal information in the acoustics, and (3) oscillation-based models are not required in order to account for the role of speaker-hearer synchronization during the decoding process. In the following we address his arguments from auditory processing viewpoint, referring to a particular phenomenological model (Ghitza, 2011) taken as a representative of the criticized oscillation-based models. In order to address Cummins' comments effectively, we start by presenting the rationale for the oscillation-based approach.

## Rationale

Speech is an inherently rhythmic phenomenon in which the acoustic signal is transmitted in “packets.” This temporal structure is presented at the cochlear output as temporal fluctuations of critical-band envelopes, with the prominent fluctuations in the range between 3 and 12 Hz (e.g., Houtgast and Steeneken, 1985). By using the term “rhythm,” we do not mean that these temporal fluctuations are periodic (in fact, they are not), but rather that there are constraints on duration and energy patterns within and across prosodic phrases, and across languages. This rhythmic variation is important for intelligibility and naturalness; speech synthesis studies, for example, have shown that listeners prefer spoken material with a natural, rhythmic structure (e.g., Schroeter, 2008; van Santen et al., 2008). Does this rhythmic property of speech reflect some fundamental property, one internal to the brain? More pointedly, are the temporal properties of spoken language the result of the evolutionary trajectory to match a cortical function, with neuronal oscillations at the core?

Temporal properties of speech are likely to be constrained not only by how fast the articulators can move, but also by how long certain phonetic constituents need to be in order for the signal to be intelligible and sound natural. The supra-segmental properties of speech, especially in view of their variability from language to language, are more likely to be the consequence of factors other than articulation. For example, the range of time intervals (40–2000 ms) associated with different levels of linguistic abstraction (phonetic feature, syllable, word, metrical foot, and prosodic phrase) may reflect temporal constraints associated with neuronal circuits in the cerebral cortex, thalamus, hippocampus, and other regions of the brain. More specifically, certain neuronal oscillations (e.g., von Stein and Sarnthein, 2000; Buzsáki, 2006) could be the reflection of both local and longer-range, trans-cortical processing. The frequency range over which such oscillators operate (0.5–80 Hz) may serve as the basis for hierarchical synchronization through which the central nervous system processes and integrates sensory information (e.g., Singer, 1999; Lakatos et al., 2005). In particular, there is a remarkable correspondence between average durations of speech units and the frequency ranges of cortical oscillations. Phonetic features (duration of 20–50 ms) are associated with gamma (>40 Hz) and beta (15–30 Hz) oscillations, syllables, and words (mean duration of 250 ms) with theta (4–8 Hz) oscillations, and sequences of syllables and words embedded within a prosodic phrase (500–2000 ms) with delta oscillations (<3 Hz).

**Figure 1:**
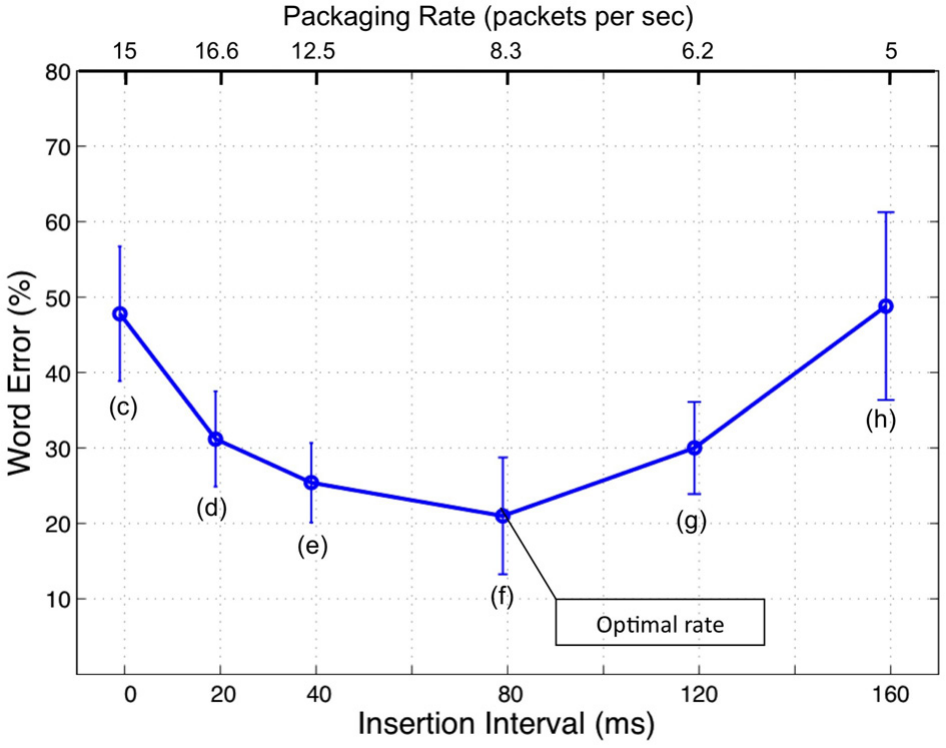
**Intelligibility of time-compressed speech with insertion of silence gaps (from Ghitza and Greenberg, 2009).** The stimuli comprised naturally spoken, semantically unpredictable sentences (i.e., no context) time-compressed by a factor of 3, with insertions of silent gaps in-between successive intervals of the compressed speech. Intelligibility was poor without insertions (about 50% word error rate) but, counter intuitively, was restored considerably by the insertion of gaps, as long as the gaps were between 20 and 120 ms. The duration of the acoustic interval was held constant (40 ms), and the sole varying parameter was the length of the inserted gap. Thus, any change in intelligibility could be attributed to the length of the inserted gap *per se*. No (purely) auditory or articulatory model can explain this behavior. The insertion of gaps was interpreted as the act of providing extra decoding time (a *cortical* factor) via “repackaging” the information stream. Furthermore, it was hypothesized that decoding time is governed by brain oscillations.

This correspondence has inspired recent hypotheses on the potential role of neuronal oscillations in speech perception (e.g., Poeppel, 2003; Ahissar and Ahissar, 2005; Ghitza and Greenberg, 2009; Ghitza, 2011; Giraud and Poeppel, 2012; Peelle and Davis, 2012). In particular, in an attempt to account for counterintuitive behavioral findings on the intelligibility of time-compressed speech as a function of “repackaging” rate (Ghitza and Greenberg, 2009; see Figure 1), a cortical computation principle was proposed according to which the speech decoding process is performed within a time-varying, hierarchical window structure *synchronized with the input* (Ghitza, 2011). The window structure was assumed to be realized by a neuronal mechanism with cascaded oscillations at the core, capable of tracking the input pseudo-rhythm embedded in the critical-band envelopes of the auditory stream. In the model, the theta oscillator is the “master” and the other oscillators entrain to theta. We stress, at the outset, that the oscillators in the array are *quasi-periodic*, as they are assumed to be capable of tracking the input pseudo-rhythm (within their biological range). Some properties of the model are worth recalling.

**Figure 2:**
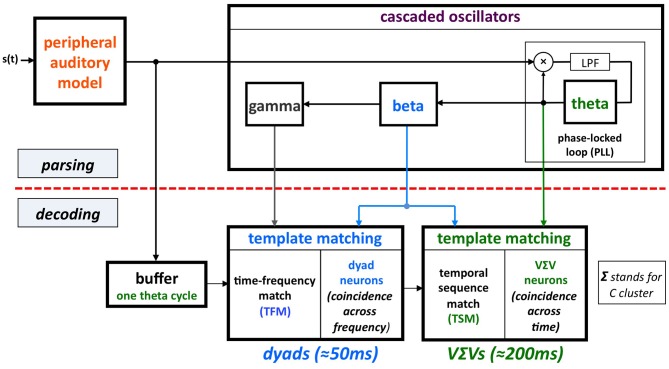
**A block diagram of the *Tempo* model.** It comprises lower and upper paths that process the sensory stream generated by a model of the auditory periphery. Conventional models of speech perception assume a strict decoding of the acoustic signal. The decoding process of Tempo conforms to this notion, linking chunks of sensory input of different durations with stored linguistic memory patterns. The additional, upper path provides *parsing* information, expressed in the form of a hierarchical window structure *synchronized with the input* and realized as an array of cascaded oscillators locked to the input syllabic rhythm. As such, the oscillators in the array are assumed to be quasi-periodic, with slowly varying frequencies. The instantaneous frequencies and relative phases of the oscillations determine the location and duration of the temporal windows that control the decoding process. The parsing path plays a crucial role in explaining the data by Ghitza and Greenberg (2009; see Figure 1). See text for details.

The model (termed *Tempo*) is shown in Figure 2. The sensory stream is processed, simultaneously, by a *parsing* path and a *decoding* path, which correspond to the upper and lower parts of Figure 2. Conventional models of speech perception assume a strict decoding of the acoustic signal^1^. The decoding path of Tempo conforms to this notion; the decoding process links chunks of sensory input of different durations with stored linguistic memory patterns. The additional parsing path, realized as an array of cascaded oscillators, determines a hierarchical window structure (location and duration) that controls the decoding process. The parsing path plays a crucial role in explaining the data by Ghitza and Greenberg (i.e., the counterintuitive U-shape performance when listening to speech uttered too fast, with or without the insertion of silence gaps) and is a helpful extension to conventional models. The key property that enables an explanation of the behavioral data is the capability of the window structure to stay synchronized with the input. The theta oscillator (the master) provides *segmental* parsing; assuming perfect tracking, a theta cycle is aligned with a segment that is often a VΣV (Σ stands for consonant cluster). (This is so because the prominent energy peaks across the auditory channels, which presumably feed the theta tracker, are associated with vowels). The windows within which the *phonetic* content is decoded (by the decoding path) are the beta cycles (entrained to theta). The role of gamma is different: it determines the time-instances at which the sensory information is sampled within the beta cycle (see Appendix in Ghitza, 2011).

**Figure 3:**
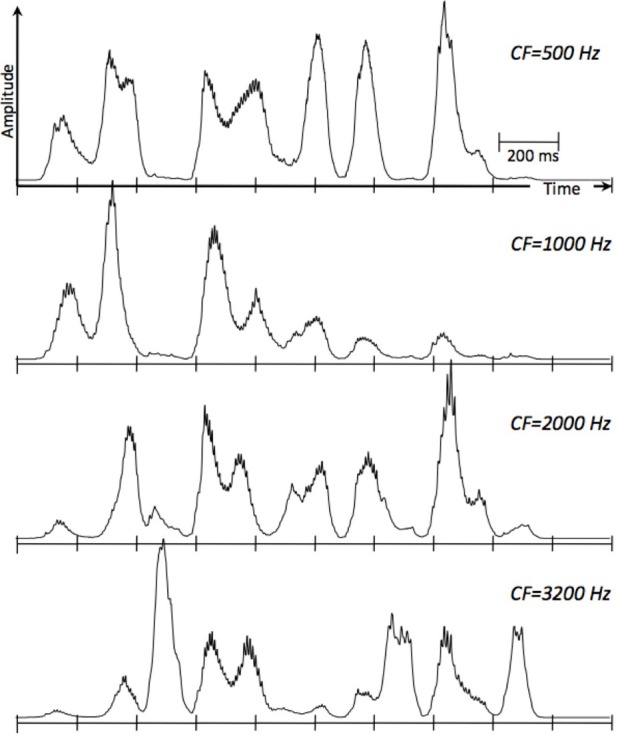
**Cochlear envelopes in terms of simulated Inner Hair Cell responses, low-pass filtered to 50 Hz, at four characteristic frequencies (CFs).** The cochlear filters are modeled as linear gammatone filters and the IHC as a half-wave rectifier followed by a low-pass filter, representing the reduction of synchrony with CF. The speech signal is roughly 2 s long (ten 200-ms long frames). The rate of the envelope fluctuations is about 4 peaks per second. Low-frequency cochlear channels mainly reflect the presence of vowels and nasals; high frequency channels mainly reflect the presence of fricatives and stop-consonants. The PLL component of Tempo (Figure 2) is assumed to be locked to the temporal fluctuations of the cortical auditory representation of the speech signal (e.g., the modulation spectrum), which is related to the cochlear response.

Three points merit discussion. First, we concur with Cummins in his observation that “the term ‘rhythm’ is used in fundamentally different ways within neuroscience—where it is treated as synonymous with ‘periodic’—and in our everyday talk of speech—where rhythm is more akin to musical rhythm, and much harder to define in an objective sense.” To avoid this ambiguity we use the term “oscillation.” Moreover, we use a *special class of oscillators*, e.g., the voltage controlled oscillator (VCO) of a phase-lock loop (PLL) system (e.g., Viterbi, 1966; Ahissar et al., 1997), which allow a gradual change in their frequency while tracking the slowly varying temporal fluctuations of the cortical auditory representation of the speech signal (see Figure 3). Second, we were aiming to offer a model for *some* critical computations in parsing and decoding speech, not a programmatic one-size-fits-all solution for all of speech comprehension. In particular, there is no attempt to posit any representational theories in Tempo. Rather, it provides the functional infrastructure to parse and decode speech in the pre-lexical level, without considering context or any lexical structure^2^. Third, the new component of Tempo, which crucially differentiates it from conventional models of speech perception, is the parsing path. The term “parsing” as employed here does not refer to the exhaustive division of the incoming speech signal into candidate constituents, or even the inference of candidate constituents from the cues in the speech signal (this is carried out by the decoding path), but rather to the function of setting a time-varying, hierarchical window structure synchronized to the input.

## Addressing cummins' arguments

A central argument in Cummins' criticism arises from a mischaracterization of the cortical function formulated by the oscillation-based models exemplified by Tempo. His assertion—that oscillation-based models do not have the structure necessary to decode the rich spectro-temporal information in the acoustics—stems from overlooking the time-varying property of the theta oscillator and the function performed by the cascaded oscillatory array as a whole, i.e., the construction of a window structure which controls the decoding process. Cummins rightly reminds us that the linguistic information (intended to be conveyed to the listener) is encoded into acoustics via a complex interaction of all articulators, not just the jaw, and asks: how could the entrainment of theta to the quasi-cyclic jaw wagging possibly decode the phonetic information in its entirety^3^? But in Tempo, the crucial role of the theta is in *parsing*: the theta oscillator tracks the critical-bands' temporal envelope modulations (in the theta range, see Figure 3)—not the wagging jaw—and the theta-driven cascaded oscillatory array results in a hierarchical window structure synchronized to the input, controlling the decoding path. The decoding process itself—i.e., linking pieces of spectro-temporal information into stored linguistic memory patterns—is performed by the decoding path circuitry, within the time-windows determined by the oscillatory array.

Cummins also questions whether oscillation-based models are required in order to account for the role of speaker-hearer synchronization during the decoding process. Referring to his own study on the role of “speech synchrony” in human-human interaction Cummins writes: “An entrainment account based on the amplitude envelope (or the jaw) as the mediating signal that yokes two systems together is fundamentally incomplete … ” And he adds: “Indeed, it was found that the amplitude envelope was neither necessary nor sufficient to facilitate synchronization among speakers (Cummins, 2009), and that synchronization depended upon a complex suite of interacting factors, among which intelligibility seemed to be the single most important (although intelligibility is not related to any single signal property).” Consequently, he advocates for a dynamical system framework in which the speaker and the listener are two elements within one system, coupled (entrained, synchronized) by rhythms. In his published work, Cummins (2009, 2011) confined his theory to a rather singular setting where the speaker and the listener are located in the same room (i.e., seeing and hearing each other). But why should this principle be restricted to this setting alone? Couldn't it hold for a telephone conversation as well? (i.e., where the listeners hear speech, artificially produced, with no access to the articulators). We, therefore, contend that Cummins' interpretation of “synchronization” is too narrow, and that our usage of the concept is in the common, less restrictive sense. We suggest that speaking in packets is the result of an evolutionary attempt to maximize information transfer to the brain of the listener, i.e., to match a cortical function. Maximizing information transfer (either for a conversation in the same room or a conversation via a telephone) is in terms of achieving maximum performance, e.g., in an intelligibility related task. Therefore, Cummins' observation—that intelligibility is the single most important facilitator of speaker/listener synchronization—cannot be separated from the crucial role of the amplitude modulations in enabling a reliable theta-driven parsing necessary for successful decoding (measured in terms of intelligibility, e.g., Ghitza, 2012).

In his closing sentence Cummins writes: “A mechanical model that treats syllable-producers as oscillators and syllable-hearers as entraining to those oscillations, seems, to this phonetician, to ignore much of the known complexity of speech as she is spoken and of speakers as they speak.” As already been noted, oscillation-based models do not assume that speech is periodic (i.e., “syllable-producers as oscillators”). Rather they use a special class of oscillators, which allow a slow change in instantaneous frequency while tracking the non-periodic temporal fluctuations of the input signal. As for the coda of the closing sentence, two levels of linguistic abstraction seems to be intertwined—the syllable and the prosodic phrase—which span two time windows, ≈200 ms long and ≈1 s long, pertaining to the theta and the delta oscillators, respectively. As already discussed, from an auditory processing point of view the theta oscillator is essential in VΣV parsing, i.e., in setting the window structure for decoding phonemes and sequences of phonemes. (This aspect of the decoding process is addressed by Tempo). The delta oscillation, in our view, plays an important role in *prosodic* parsing, which pertains to sequences of words hence tapping contextual effects. As such, we believe that the delta oscillator interacts with the theta in a top-down fashion. The manner by which this process is carried out cortically is yet to be formulated.

## The theta-syllable

We conclude by expanding on an important follow-up comment raised by Cummins, at the heart of the search for the acoustic correlate of the syllable. Cummins asserts: “The syllable is a construct that is central to our understanding of speech,” but he adds: “The apparent facility with which the syllable is employed in many accounts belies an important observation: syllables are not readily observable in the speech signal … Even competent adult English speakers may have difficulty counting syllables in a given utterance.” A corollary to this observation is that a consistent acoustic correlate to the syllable is hard (if not impossible) to define. So, in spite of the important role the syllable plays in our understanding of how basic speech units are *produced*, a question arises: in view of its inherently ambiguous definition in the acoustics, should the syllable play a central role in our understanding of how speech is *perceived*?

Of course, hearers are capable of isolating units like syllables or phones: listeners can perform remarkably well in laboratory tasks related to syllable recognition (e.g., discrimination or classification tasks). However, our focus is in understanding of how spoken language is decoded in everyday speech. What do oscillation-based models tell us about how fluent speech may be parsed and decoded?

Indeed, for single isolated words, oscillator-based models do not provide any additional insights into our understanding of how sub-word units are decoded. This is so because the duration of the stimulus is too short to allow entrainment, resulting in an oscillatory array in idle mode and a system reduced to the conventional model (i.e., the decoding path in Tempo). In contrast, everyday speech is long, enough to allow entrainment. Indeed, such signal exhibits substantial irregularity in timing, e.g., in the form of hesitation and disfluency. How such irregularities affect the performance of the parsing path? Tempo provides a framework to a reasonable explanation of the manner by which the cortical receiver handles this difficulty; when the input rhythm is unsettled the theta oscillator (and hence the entire array) is idling at its core frequency (say at mid range), ready to reenter the tracking mode. Once in tracking mode, the parsing path forms a window structure synchronized with the input, comprising windows within a time span of a theta cycle (aligned with a VΣV segment)^4^. In light of the role of the theta oscillator in parsing, an auditory-driven unit of speech information emerges with a non-ambiguous acoustic correlate:

**Definition**: The *theta-syllable* is a theta-cycle long speech segment located between two successive vocalic nuclei.

Three points are worth noting. First, given the prominence of vocalic nuclei in the presence of environmental noise the theta-syllable is also *robustly* defined. Vocalic nuclei alone, however, are insufficient for defining the syllable boundaries (even though they provide audible cues that correspond to syllable “centers”). Second, the theta-syllable is invariant under time scale modifications that result in intelligible speech. When listening to time-compressed speech that is intelligible, the cortical theta is in sync with the stimulus. Thus, the speech segment that corresponds to a theta cycle is the time-compressed version of the corresponding original VΣV segment. Third, although outside the scope of Tempo, it is relevant to recall the *lexical function* of word segmentation. Based upon the performance of adult hearers in a speech segmentation task while listening to fluent speech, Cutler (1994; see also Cutler, 2012) concluded that—in everyday speech—the smallest linguistic meaningful units are *words*. Intriguingly, she added, “data plainly indicate that rhythm in the input makes segmenting speech a breeze.”^5^ Her observation raises the possibility of a rhythm-based approach to word segmentation, coupled in a natural way with the pre-lexical, oscillator-based models a-la Tempo.

### Conflict of interest statement

The author declares that the research was conducted in the absence of any commercial or financial relationships that could be construed as a potential conflict of interest.

## Footnotes

^1^Conventional models of speech perception rely, almost exclusively, on the acoustics of the speech itself. Phones are identified first, and the ordered sequence of identified phones results in a pointer to the word lexicon (e.g., Marslen-Wilson, 1987; Luce and McLennan, 2005; Stevens, 2005).

^2^The speech material of Ghitza and Greenberg comprised of naturally spoken, *semantically unpredictable* sentences (i.e., sentences without context).

^3^Being posed by a phonetician the question is narrow in scope. From an auditory processing point of view, “quasi-cyclic jaw wagging” should be substituted with “amplitude modulations of the signal.” After all, the mechanism by which the acoustics is generated—being the human speech production apparatus or an electronic speech synthesis device—is of no relevance here.

^4^Recall the necessity of the parsing path, in light of the crucial role it plays in accounting for the counterintuitive behavioral data of Ghitza and Greenberg.

^5^Cutler's usage of “rhythm” attends the variation in rhythmic characteristics across languages [e.g., stress timing (English, Swedish) vs. syllable timing (French, Spanish)].

## References

[B1] Ahissar E. Ahissar M. (2005). Processing of the temporal envelope of speech, in The Auditory Cortex. A Synthesis of Human and Animal Research, Chap. 18, eds Konig R. Heil P. Bundinger E. Scheich H.(Mahwah, NJ: Lawrence Erlbaum), 295–313

[B2] Ahissar E. Haidarliu S. Zacksenhouse M. (1997). Decoding temporally encoded sensory input by cortical oscillations and thalamic phase comparators. Proc. Natl. Acad. Sci. U.S.A. 94, 11633–11638 9326662 10.1073/pnas.94.21.11633 PMC23560

[B3] Buzsáki G. (2006). Rhythms of the Brain. New York, NY: Oxford University Press

[B4] Cummins F. (2009). Rhythm as entrainment: the case of synchronous speech. J. Phon. 37, 16–28

[B5] Cummins F. (2011). Periodic and aperiodic synchronization in skilled action. Front. Hum. Neurosci. 5:170 10.3389/fnhum.2011.00170 22232583 PMC3248675

[B6] Cummins F. (2012). Oscillators and syllables: a cautionary note. Front. Psychol. 3:364 10.3389/fpsyg.2012.00364 23060833 PMC3464477

[B7] Cutler A. (1994). The perception of rhythm in language. Cognition 50, 79–81 8039376 10.1016/0010-0277(94)90021-3

[B8] Cutler A. (2012). Native Listening: Language Experience and the Recognition of Spoken Words. Cambridge, MA: MIT Press

[B9] Ghitza O. (2011). Linking speech perception and neurophysiology: speech decoding guided by cascaded oscillators locked to the input rhythm. Front. Psychol. 2:130 10.3389/fpsyg.2011.00130 21743809 PMC3127251

[B10] Ghitza O. (2012). On the role of theta-driven syllabic parsing in decoding speech: intelligibility of speech with a manipulated modulation spectrum. Front. Psychol. 3:238 10.3389/fpsyg.2012.00238 22811672 PMC3397378

[B11] Ghitza O. Greenberg S. (2009). On the possible role of brain rhythms in speech perception: intelligibility of time-compressed speech with periodic and aperiodic insertions of silence. Phonetica 66, 113–126 10.1159/000208934 19390234

[B12] Giraud A. L. Poeppel D. (2012). Cortical oscillations and speech processing: emerging computational principles and operations. Nat. Neurosci. 15, 511–517 10.1038/nn.3063 22426255 PMC4461038

[B13] Houtgast T. Steeneken H. J. M. (1985). A review of the MTF concept in room acoustics and its use for estimating speech intelligibility in auditoria. J. Acoust. Soc. Am. 77, 1069–1077

[B14] Lakatos P. Shah A. S. Knuth K. H. Ulbert I. Karmos G. Schroeder C. E. (2005). An oscillatory hierarchy controlling neuronal excitability and stimulus processing in the auditory cortex. J. Neurophysiol. 94, 1904–1911 10.1152/jn.00263.2005 15901760

[B15] Luce P. A. McLennan C. (2005). Spoken word recognition: the challenge of variation, in The Handbook of Speech Perception, eds Pisoni D. B. Remez R. E. (Malden, MA: Blackwell Publishing), 591–609

[B16] Marslen-Wilson W. D. (1987). Functional parallelism in spoken word-recognition. Cognition 25, 71–102 10.1016/0010-0277(87)90005-9 3581730

[B17] Peelle J. E. Davis M. H. (2012). Neural oscillations carry speech rhythm through to comprehension. Front. Lang. Sci. 3:320 10.3389/fpsyg.2012.00320 22973251 PMC3434440

[B18] Poeppel D. (2003). The analysis of speech in different temporal integration windows: cerebral lateralization as ‘asymmetric sampling in time.’ Speech Commun. 41, 245–255

[B19] Schroeter J. (2008). Basic principles of speech synthesis, in Handbook of Speech Processing, eds Benesty J. Sondhi M. M. Huang Y. (Berlin: Springer-Verlag), 413–428

[B20] Singer W. (1999). Neuronal Synchrony: a versatile code for the definition of relations? Neuron 24, 49–65 10.1016/S0896-6273(00)80821-1 10677026

[B21] Stevens K. (2005). Features in speech perception and lexical access, in The Handbook of Speech Perception, eds Pisoni D. B. Remez R. E. (Malden, MA: Blackwell Publishing), 125–155

[B22] van Santen J. P. H. Mishra T. Klabbers E. (2008). Prosodic processing, in Handbook of Speech Processing, eds Benesty J. Sondhi M. M. Huang Y. (Berlin: Springer-Verlag), 471–487

[B23] Viterbi A. J. (1966). Principles of Coherent Communication. New York, NY: McGraw-Hill

[B24] von Stein A. Sarnthein J. (2000). Different frequencies for different scales of cortical integration: from local gamma to long range alpha/theta synchronization. Int. J. Psychophysiol. 38, 301–313 10.1016/S0167-8760(00)00172-0 11102669

